# Complications of the Bacillus Calmette-Guerin vaccine as an early warning sign of inborn errors of immunity: a report of 197 patients

**DOI:** 10.3389/fimmu.2024.1477499

**Published:** 2024-12-05

**Authors:** Mohammad Reza Fazlollahi, Ali Goudarzi, Maryam Nourizadeh, Zahra Alizadeh, Shaghayegh Tajik, Mohsen Badalzadeh, Shokouh Azam Sarafzadeh, Maryam Mahlooji Rad, Zeinab Adab, Leila Moradi, Anahita Razaghian, Nastaran Sabetkish, Zahra Pourpak, Mostafa Moin

**Affiliations:** ^1^ Immunology, Asthma and Allergy Research Institute, Tehran University of Medical Sciences, Tehran, Iran; ^2^ Children’s Medical Center, Pediatrics Center of Excellence, Tehran University of Medical Sciences, Tehran, Iran; ^3^ Division of Allergy and Clinical Immunology, Department of Pediatrics, Hakim Children Hospital, Tehran University of Medical Sciences, Tehran, Iran

**Keywords:** Bacillus Calmette-Guérin vaccine, inborn errors of immunity, BCGosis, BCGitis, primary immunodeficiencies

## Abstract

**Background:**

According to the WHO’s recommendation for developing countries, Bacillus Calmette-Guerin (BCG) vaccination has been implemented in some countries as part of national vaccination programs at birth. Although it is generally considered safe, some complications may occur; including BCGitis (local) or BCGosis (systemic), ranging from mild like local abscesses to fatal impediments like osteomyelitis and disseminated BCG infection. This study aimed to determine the spectrum of inborn errors of immunity (IEI) in BCG-vaccinated neonates experiencing local or systemic complications.

**Methods:**

In this cross-sectional study, we investigated Iranian children referred to the Immunology, Asthma, and Allergy Research Institute (IAARI) between 2007-2023 for suspected immunodeficiency. Medical history was recorded, and primary screening tests for immunodeficiency were conducted for all cases. For suspected cases, more advanced immunologic investigations were performed to reach a definitive diagnosis. Furthermore, the study incorporated the documented genetic findings of the patients under investigation. All patients with inborn error of immunity who had a history of BCG vaccine complications within the first year of vaccination were enrolled in the study.

**Results:**

We investigated 3,275 cases suspected of IEI, identifying197 patients with both IEI and BCG vaccine complications. Among these, 127 (64.5%) were male. Symptoms began at or before 3 months of age in 64.8% of the cases, and parental consanguinity was reported in 79.2%. Genetic diagnoses were confirmed in 108 patients. Of the 197 patients, 108 (54.8%) had BCGitis, while 89 (45.2%) experienced systemic complications (BCGosis). A family history of IEI, BCG-related complications, and unexplained deaths were observed in 20.3%, 12.2%, and 29.9% of cases, respectively. Furthermore, 46.2% had at least one of these three risk factors in their history.

**Conclusions:**

Early BCG vaccine complications may indicate an underlying immunodeficiency, particularly when there is a positive family history of BCG complications, immunodeficiency, or unexplained deaths. Nation-wide vaccination protocols should address this issue by delaying inoculation to allow for immunological screening of suspected immunodeficient patients, thereby preventing BCG vaccine-related morbidity and mortality.

## Introduction

The Bacillus Calmette-Guérin (BCG) vaccine is administered to all newborns in some countries, as it is believed to provide 64–78% protection against severe forms of tuberculosis, including meningeal and miliary tuberculosis (1). The World Health Organization (WHO) recommends administrating the BCG vaccine at birth as a policy to prevent severe forms of tuberculosis in countries where the disease is endemic. It is estimated that around 100 million children receive the BCG vaccine annually (2). As part of Iran’s national vaccination program, all newborns are given an intradermal injection of 0.05 mL of a live, attenuated strain of *Mycobacterium Bovis* (3). The predominant strain of the BCG vaccine used in Iran is the “Pasteur 1173P2 strain” which is administered to all newborns as part of the national tuberculosis vaccination program (4).BCG vaccination triggers a delayed cell-mediated hypersensitivity reaction, typically occurring 4 to 8 weeks post-inoculation (5).

A normal cutaneous reaction presents as a 5 to 15-millimeter (mm) red, indurated area at the injection site. By 6–10 weeks, a crust forms over the induration, which eventually falls off, leaving a 3–7 mm scar (6). Although generally safe, the vaccine can cause complications, including local infections (BCGitis) and systemic complications like disseminated BCG infection (BCGosis). The incidence of BCG-related complications depends on various factors, including vaccination dose, storage conditions, strains used, injection technique, age, demographics, immune status of the recipient, and the method of vaccine production. Local complications are usually self-limiting, occurring at a rate of 4-30 per 1,000 vaccinated newborns (1, 6, 7). Systemic complications, or BCGosis, are more severe and potentially fatal, primarily affecting individuals with weakened immune system but also occasionally occurring in healthy individuals (8). BCGosis is rare, with an incidence of 0.1 to 4.3 per million vaccinated newborns and a mortality rate of 71%, increasing to 83% in immunocompromised patients. It can manifest between 1 and 12 months after vaccination (9).

Inborn errors of immunity (IEI) previously referred to “primary immunodeficiencies”, are a group of inherited conditions characterized by impaired immune responses. These conditions can manifest in various ways, including recurrent infections, malignancies, and immune dysregulation. With an estimated prevalence of 1:1200, IEI may affect approximately 1% of the population. Given the broad spectrum of clinical presentations, early diagnosis and treatment are critical. One key warning sign of IEI is complications from vaccines, particularly those associated with the BCG vaccine. For instance, BCGosis, a severe granulomatous disease triggered by the BCG vaccine, can signal underlying IEI such as chronic granulomatous disease (CGD) or severe combined immunodeficiency (SCID). Recognizing these complications early enable targeted treatment and better outcomes for patients with IEI (10, 11).

This study aimed to evaluate patients with suspected immunodeficiency who developed BCG vaccine-related complications, with the goal of emphasizing BCG vaccine complications as an important indicator of underlying IEI.

## Materials and methods

### Patients

This is an institutional-based and descriptive cross-sectional study on the patients referred to the Immunology, Asthma, and Allergy Research Institute (IAARI) for suspected immunodeficiency with concomitant BCG vaccine complications up to 12 months of age during 2007 to 2023. This study was approved by the IAARI’s Ethics committee. A specific questionnaire was completed for all patients, containing demographic data, information on consanguinity, the onset of BCG complications, clinical symptoms, therapeutics interventions, duration of treatment, and family history of immunodeficiency diseases, BCG vaccine complications, or unexplained deaths.

Patients with local complications (BCGitis) are defined by the presence of axillary, cervical, or supraclavicular lymphadenitis on the same side of the vaccination site. The spread of the BCG infection (BCGosis) is characterized by symptoms such as fever or sub-febrile status, weight loss, stunted growth, and the involvement of two or more areas beyond the injection site. These areas can include lymph nodes, skin, soft tissues, lungs, liver, spleen, and bones. According to the European Society for Immunodeficiencies (ESID) (12), a definite diagnosis of disseminated BCG infection (BCGosis) is confirmed by detecting *Mycobacterium bovis* through standard PCR or culture of patient samples, along with histopathological evidence of granulomatous inflammation.

The presence of the *Mycobacterium tuberculosis* complex without differentiation of *Mycobacterium bovis*, along with granulomatous histopathological changes, indicates a probable diagnosis of BCGosis. On the other hand, histopathological evidence of granulomas without detection of specific *Mycobacterium* substrains suggests a possible diagnosis (13, 14). In this study, patients with possible, probable, or definite BCG-related complications were classified as having BCGosis, Primary and advanced immunological tests were also conducted to diagnose IEI based on ESID criteria for immunodeficiency (12). Additionally, the genetic findings of the patients were analyzed.

Anti-tuberculosis (anti-TB) treatment was administered to patients diagnosed with disseminated BCG disease (BCGosis), following national and WHO guidelines (15, 16). The treatment regimen included a combination of isoniazid, rifampicin, ethambutol, and streptomycin. In cases of Mendelian Susceptibility to Mycobacterial Disease (MSMD), second-line anti-mycobacterial agents (such as fluoroquinolones, aminoglycosides, and macrolides) were also used. Supportive immunotherapy, such as interferon-gamma (IFN-γ), was provided for patients with CGD and MSMD. Hematopoietic stem cell transplantation (HSCT) was recommended for certain cases, particularly for patients with SCID, combined immunodeficiency (CID), and CGD.

### Statistical analysis

IBM SPSS statistics software version 22 (IBM Corp, Armonk, NY, USA) was employed to analyze the data. Frequencies and percentages were calculated for qualitative variables.

## Results

From January 2007 to January 2023, 3,275 patients were referred to IAARI for suspected primary immunodeficiency. Among these, 247 presented with complications related to the BCG vaccine, and 197 patients who met the inclusion criteria for immunodeficiency were enrolled in the study over the 15-year period. Of the enrolled patients, 127 (64.5%) were male and 70 (35.5%) were female. Symptoms began at ≤3 months of age in 64.8% of the cases, while 35.2% developed symptoms between 3 and 12 months. Parental consanguinity was reported in 79.2% of cases, with 55.3% being first cousins and 23.9% second or third cousins. Family histories revealed BCG vaccine complications in 12.2%, primary immunodeficiency in 20.3%, and unexplained death in 29.9% of patients, having at least one of three risk factors in their history.

Of the 197 patients, 108 (54.8%) had local complications (BCGitis), while 89 (45.2%) experienced systemic complications (BCGosis). The median age of diagnosis of IEI was 21.5 months in the BCGitis and 6 months in the BCGosis groups.

**Figure 1:**
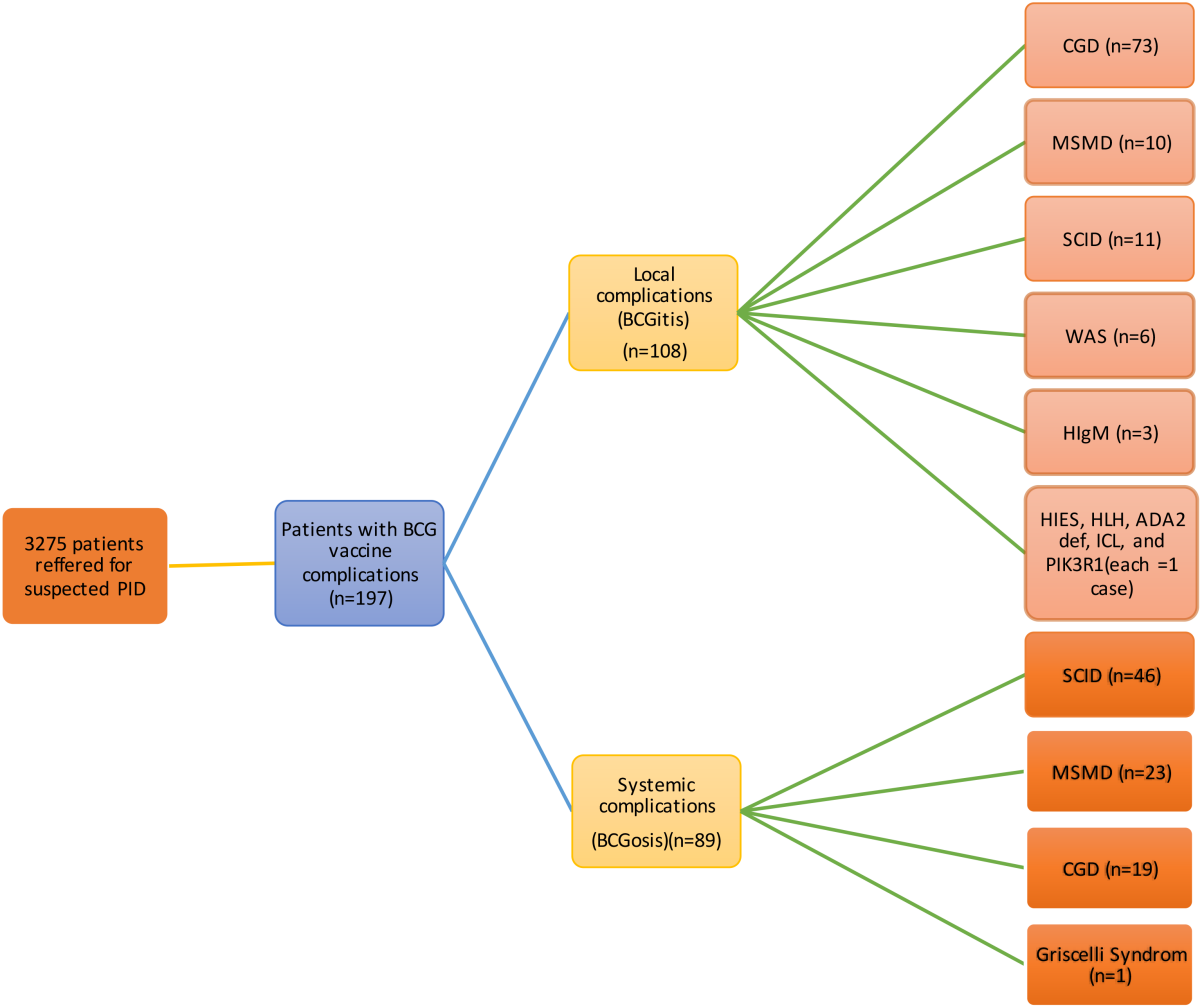
Complications of BCG in vaccinated patients (n=197). BCG, Bacillus Calmette-Guerin; CGD, Chronic granulomatous disease; MSMD, Mendelian susceptibility to mycobacterial diseases; SCID, severe combined immunodeficiency; WAS, Wiskott-Aldrich syndrome; HIgM, Hyper IgM; HIES, Hyper Immunoglobulin E syndrome; HLH, Hemophagocytic lymphohistiocytosis; DADA2, Adenosine Deaminase 2 Deficiency; ICL, Idiopathic CD4 lymphocytopenia; PIK3R1, Phosphoinositide-3-Kinase Regulatory Subunit 1.

The most common IEI were CGD (46.7%, 92 cases), SCID (28.9%, 57 cases), and MSMD (16.8%, 33 cases). Less common IEI included Wiskott-Aldrich syndrome (WAS, 3%, 6 cases), hyper Immunoglobulin M syndrome (HIM, 1.5%, 3 cases), Griscelli syndrome, Hyper Immunoglobulin E syndrome (HIES), Hemophagocytic lymphohistiocytosis (HLH), Adenosine Deaminase 2 Deficiency (DADA2), Idiopathic CD4 lymphocytopenia (ICL), and PIK3R1 mutation (each with 0.5% or 1 case). The frequency of various IEI categorized based on their propensity to cause local or systemic complications, is presented in **Figure 1**. A total of 108 patients had confirmed genetic mutations, summarized in **Table 1**.

**Table 1:** Genetic results of inborn errors of immunity (IEI) patients with BCG vaccine complications.

IEI patients	Gene types	Number (patients)	BCGitis (patients)	BCGosis (patients)
**CGD**	*CYBB* (X-CGD)	18	13	5
	*CYBA* (AR-CGD)	28	21	7
	*NCF1* (AR-CGD)	17	14	3
	*NCF2* (AR-CGD)	3	2	1
**Total CGD**		**66**	**50**	**16**
**SCID**	*RAG2*	11	2	9
	*ADA*	3	–	3
	*IL2RG*	1	1	–
	*DCLRE1C*	1	–	1
	*IL7R*	1	–	1
	*LAT*	1	–	1
**Total SCID**		**18**	**3**	**15**
**WAS**	*WAS*		4	–
**MSMD**	*IL12RB1*	10	2	8
	*IL12B*	2	–	2
	*TYK2*	1	1	–
**Total MSMD**		**13**	**3**	**10**
**Hyper IgM**	*PIK3CD*1	1	1	–
	*CD40*	1	1	–
**Total Hyper IgM**		**2**	**2**	
**Others**	*STAT3*	1	1	–
	*ADA2def.*	1	1	–
	*CD4def.*	1	1	–
	*PIK3R1*	1	1	–
	*RAB27A*	1	1	–
**Total others**		**5**	**5**	
**Total IEI Patients**		**108**		

CGD, Chronic Granulomatous Disease; SCID, Severe Combined Immunodeficiency; WAS, Wiskott-Aldrich Syndrome; MSMD, Mendelian Susceptibility to Mycobacterial Disease; Hyper IgM, hyper IgM Syndrome; IEI, Inborn Error of Immunity.

**Figure 2:**
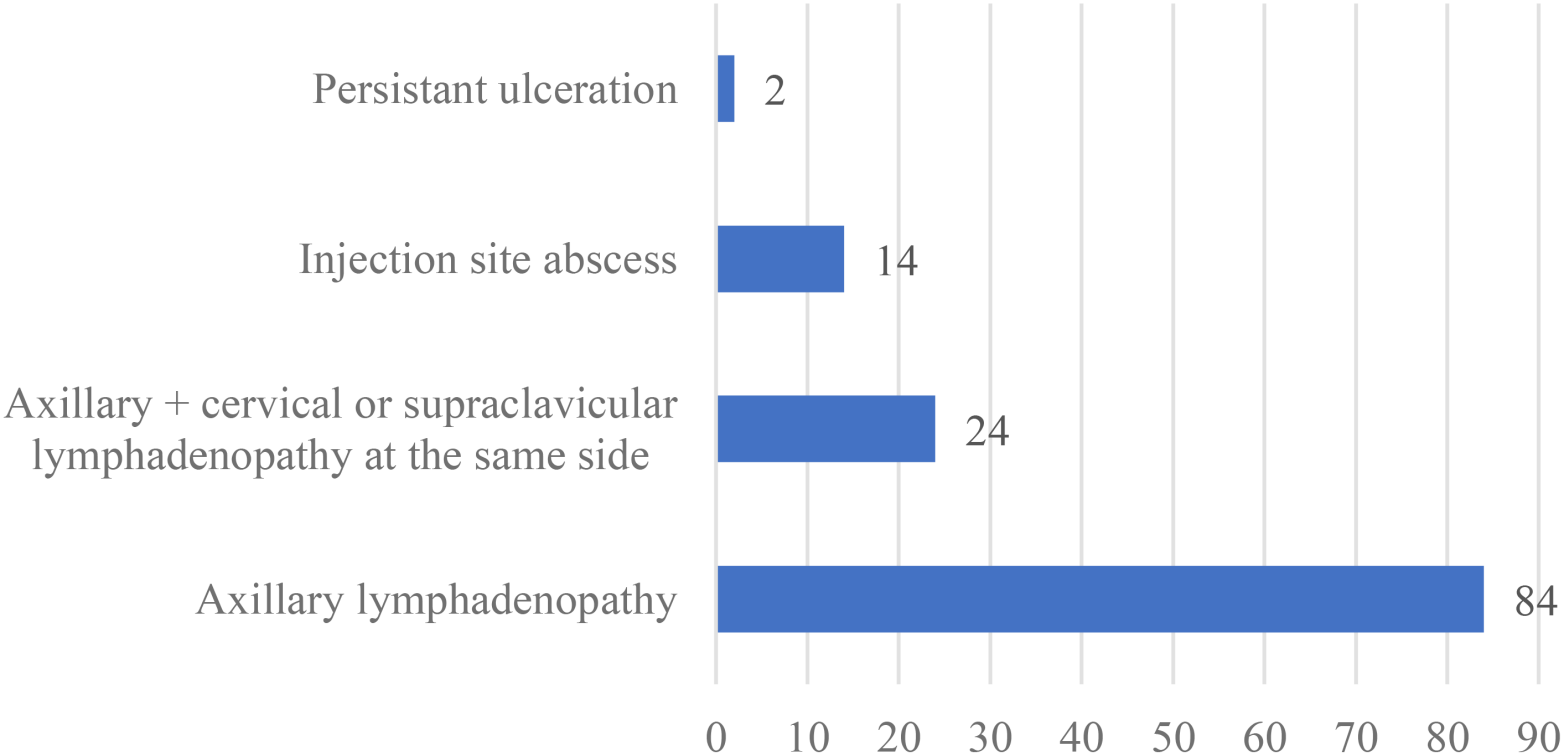
The frequency of different manifestations in 108 patients with BCGitis in this study.

Clinical symptoms of BCG-related complications were categorized by the site of involvement. Among 108 patients with local complications, 84 (77.8%) cases had axillary lymphadenopathy, while 24 (22.2%) cases presented with axillary lymphadenopathy along with cervical or supraclavicular lymphadenopathy. Injection site abscesses were reported in 14 cases (13%), and persistent ulceration in 2 cases (1.9%). No cases of keloid, lupoid reactions or osteitis were observed (**Figure 2**).

**Figure 3:**
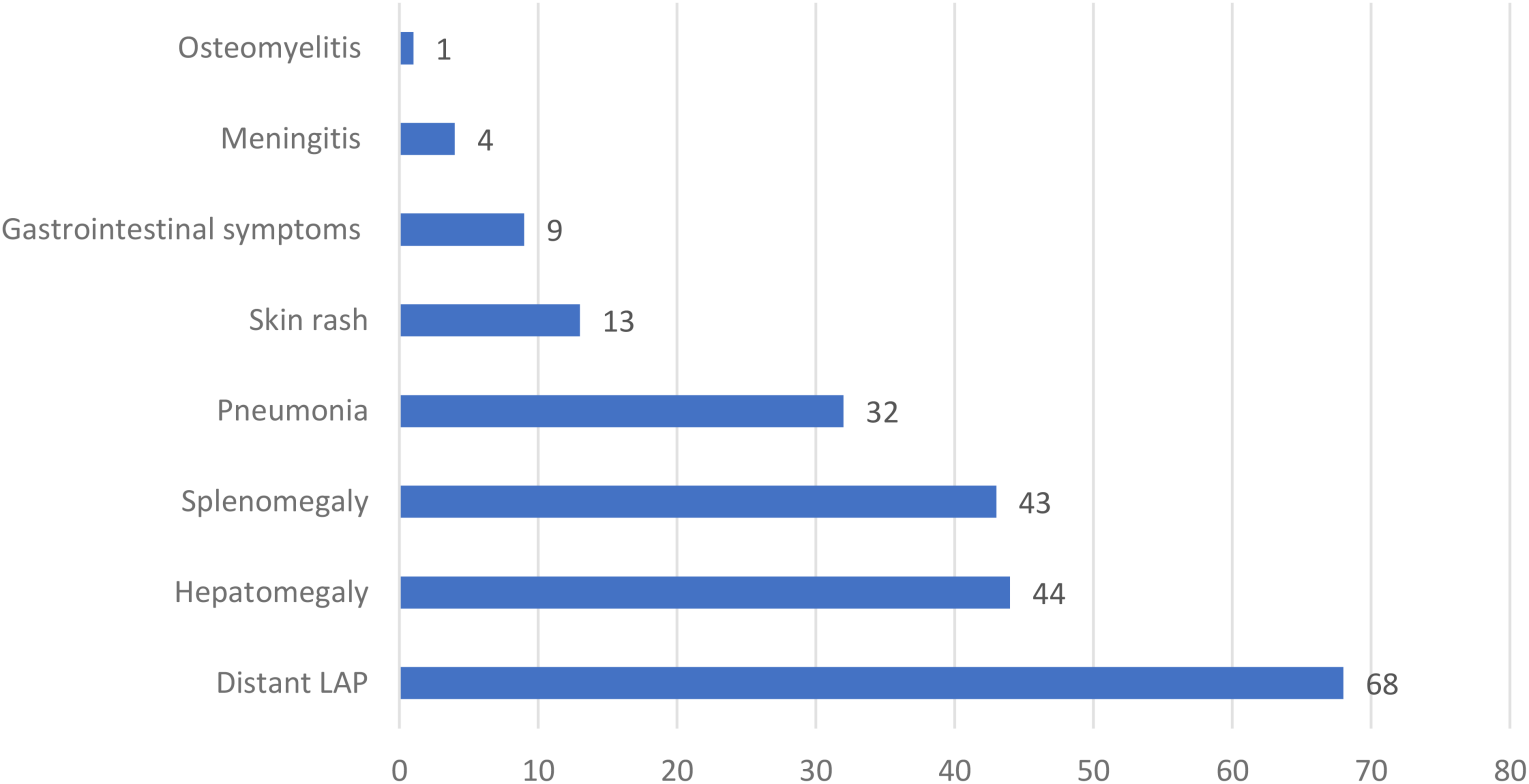
The frequency of different manifestations in 89 patients with systemic complications of BCG vaccine in this study. LAP, Lymphadenopathy.

In the 89 patients with BCGosis, 57 (64%) had axillary lymphadenopathy, and 28 (31.5%) had axillary lymphadenopathy along with cervical or supraclavicular lymphadenopathy. Other symptoms included distant lymph node involvement (76.4%, 68 patients), hepatomegaly (49.4%, 44 patients), splenomegaly (48.3%, 43 patients), pneumonia (36%, 32 patients), gastrointestinal symptoms (10.1%, 9 patients), skin rash (14.6%, 13 patients), meningitis (4.5%, 4 patients), and osteomyelitis (1.1%, 1 case) (**Figure 3**).

Of the 108 patients with BCGitis, 40 received dual-drug therapy with isoniazid and rifampicin, 46 patients improved spontaneously without treatment, and information on 22 patients was unavailable. Among the 40 patients treated, 33 improved, while 7 died. Of the 46 untreated patients, 3 died from other vaccine-related complications.

Of the 89 patients with systemic complications, 65 received treatment, while information on 24 cases was unavailable. Among those treated, 30 survived, and 35 died. The causes of death were attributed to both opportunistic infections associated with their conditions and vaccine complications.

In total, 66 out of the 197 patients (33.5%) with BCG-related complications died. Among the 108 patients with local complications, 14 deaths (13%) were recorded, all of which occurred in patients with underlying immunodeficiencies: 2 with CGD, 11 with SCID, and 1 with WAS.

The mortality rate was significantly higher in patients with disseminated or systemic complications, with 52 out of 89 patients (58.4%) dying, despite receiving anti-TB treatment. SCID accounted for 84.6% of the death among those with systemic complications.

## Discussion

This study examined 197 Iranian patients with local or systemic complications related to the BCG vaccine. It was observed that 79.2% of the patients were from consanguineous marriages, a rate significantly higher than the general consanguinity rate in Iran, which is 38.6%. This finding is consistent with previous reports showing a consanguinity rate of 65.6% among Iranian families with patients diagnosed with IEI. These results suggest a potential link between consanguinity and the development of IEI (17, 18). Given that IEI is a hereditary condition, individuals born to parents in consanguineous marriages may face an increased risk of developing these diseases and experiencing BCG-related complications.

One potential warning sign of IEI is the occurrence of severe complications following BCG vaccination, which is widely used to prevent tuberculosis. This raises the possibility that affected individuals may have an underlying immunodeficiency (19). Our study revealed a broad range of IEI among patients with BCG-related complications, with CGD being the most prevalent (46.7%), followed by SCID (28.9%), and MSMD (16.8%). In contrast, a recent systematic review of 1,691 IEI cases found that 41.5% were linked to BCG vaccine complications, with MSMD (42.4%), SCID (24.8%), and CGD (19.9%) being the most common underlying conditions (19). These differences in prevalence rates may be attributed to variations in study design, population characteristics, and methodology. Nonetheless, both studies emphasize the importance of considering IEI in patients who experience BCG-related complications. Early recognition of these complications can lead to prompt diagnosis and implementation of targeted therapies, improving outcomes for patient with IEI.

Lotte et al. conducted a comprehensive review of approximately 10,000 cases of adverse reactions to the BCG vaccine and found a significant incidence of complications, including suppurative lymphadenitis, keloid formation, and abscesses (20). Similarly, in our study, all 108 patients with BCGitis exhibited lymphadenopathy (100%), and 14 patients (13%) developed injection site abscesses.

In a 1997 literature review by Talbot et al., 28 patients with disseminated BCG infection were reported, and 24 (85.7%) of these patients were found to have IEI (21). A study from Japan reported that 39 cases of BCGosis (33.3%) were associated with IEI (22), while Aleami et al. found that approximately half of 34 patients with disseminated BCG infection had IEI (1).

In a case series and literature review, Shahmohammadi et al. identified that 10 out of 17 cases of disseminated BCG infection had underlying IEI (23).These studies, including our own, underscore the potential risks posed by the BCG vaccine in patients with undiagnosed immunodeficiency.

Talbot et al. also found that the most common symptoms among 28 patients with disseminated BCG infection were fever, lymphadenopathy, weight loss, failure to thrive (FTT), and hepatosplenomegaly (21). Additionally, an analysis of 26 neonates with disseminated tubercular lesions following BCG vaccination identified the liver, lymph nodes, spleen, and lungs as the most commonly involved organs (24). Aelami et al. reported that in 34 cases of BCGosis, prevalent symptoms included fever, axillary lymphadenopathy, hepatosplenomegaly, growth retardation, and distant lymphadenopathy. Our findings regarding BCG-related symptoms align with those reported in previous studies, highlighting similar clinical presentations and complications.

The mortality rate in BCGosis cases has been reported to vary across different studies: 58.8% (1), 40% (25), 71% (21), 73% (26), and 35% (23). In our study, the mortality rate among BCGosis patients was 58.4%. The outcomes associated with BCG-related complications can differ significantly depending on the underlying immunodeficiency. These reports underscore the high mortality risk linked to BCGosis in patients with immunodeficiencies, highlighting the need for careful pre-vaccination screening for IEI.

It is important to note that while hematopoietic stem cell transplantation (HSCT) is the only curative treatment for patients with SCID, BCG-related complications can lead to severe outcomes, including death, even following HSCT (27), However, a study by Barkai et al. found that BCG complications did not adversely impact HSCT outcomes, in fact, SCID patients without BCG complications had worse prognoses (28).

Further research in patients with CGD indicates that while X-linked chronic granulomatous disease (X-CGD) patients tend to experience severe infections at a younger age compared to those with autosomal recessive CGD (AR-CGD), BCG infections are frequent in both AR-CGD and X-CGD patients, regardless of genotype or mutation type (29). Our findings in CGD patients mirrored these results, with frequencies of BCGitis and BCGosis at 72.2% and 27.7%, respectively, in X-CGD patients, and 77.1% and 22.9%, respectively, in AR-CGD patients.

In this study, fewer than half of the patients with local complications received anti-TB treatment. The lack of anti-TB drug administration in some of these patients was largely due to the inability to achieve a definitive diagnosis of the disease at the onset of BCGitis. At that time, the diagnosis of IEI had not been confirmed, as the median age of diagnosis was 21.5 months. Although some of these patients experienced spontaneous improvement, the eventual appearance of other symptoms led to a definitive diagnosis of IEI. This emphasizes the importance of taking any vaccine complication seriously, ensuring timely diagnostic and therapeutic measures are implemented. After a definitive diagnosis of IEI, it is crucial that patients receive anti-TB treatment, especially those with MSMD and SCID (28, 30). Furthermore, patients diagnosed with SCID or CID who had received the BCG vaccine prior to transplantation should begin dual anti-tuberculosis prophylaxis, including isoniazid and rifampin (31). Both this study and previous reports indicate that most complications related to the BCG vaccine occur in individuals with IEI. Hence, vaccinating neonates who are immunocompromised is contraindicated (32).

Since BCG vaccination is a mandatory program in Iran, as well as in several other countries (33), genetic counseling and prenatal diagnosis can play a vital role in informed decision-making and early intervention for affected families (34). Newborn screening represents one of the most effective preventive strategies to reduce BCG complications in IEI patients. Recent advancements in screening techniques such as T-cell receptor excision circles (TREC) assays have been developed specifically for SCID patients (35–37). Moreover, training healthcare professionals to avoid administering live vaccines like BCG to patients with a family history of primary immunodeficiency, vaccine complications, or unexplained death is crucial. The timing of vaccination has also been emphasized in various studies. Burl et al. found that administering the BCG vaccine at 4.5 months elicits a similar immune response to *Mycobacterium tuberculosis* as vaccination at birth (38, 39). Consequently, Japan vaccinates infants at three months of age given the rarity of disseminated BCG infection (12).

Additionally, Ildirim et al. reported that delayed vaccination is both more effective and associated with fewer complications (40). Pabst et al. recommended administering the vaccine nine months after birth (41). In 2005, Sweden shifted its BCG vaccination age from birth to six months after observing increased cases of BCGitis in neonates. Although the shift resulted in a rise in atypical mycobacterial diseases, the policy remains in place (42). Considering the range of BCG-related complications, especially the most severe cases in children with IEI, Al Waili et al. suggest delaying the BCG vaccine to six months to allow for the identification of IEI (29).

Our findings revealed that 46.2% of patients had a positive family history of BCG vaccine complications, IEI, or unexplained death. These risk factors should be incorporated into vaccination protocols, considering postponement or contraindication for BCG administration in infants. While such warnings are present in Iran’s national vaccination protocol, further training is necessary for healthcare professionals in maternity wards to ensure strict adherence to these Determining the optimal time for vaccination should take into account both the prevalence of tuberculosis and IEI, along with their respective complications.

Although delaying BCG vaccination has been emphasized in prior studies, shifting the administration schedule from birth to six months does not completely prevent complications in some IEI patients. For example, in a cohort study, the median age of onset for systemic complications in patients with combined immunodeficiency was seven months (43).

Therefore, based on nationwide vaccination protocols, TB incidence rates, and healthcare resources, a comprehensive approach is recommended:

1. Neonatal IEI screening: Implement TREC and KREC (κ-deleting recombination excision circle) screening to identify at-risk infants before BCG vaccination.
2. Immunological screening tests: Conduct these for high-risk infants, such as those with: A family history of IEI or severe BCG vaccine complications and/or recurrent infections or unexplained deaths in their family.
3. Delayed BCG Vaccination: Postpone BCG vaccination from birth to 3-6 months for all children.

This approach aims to safeguard vulnerable populations while optimizing the effectiveness of the BCG vaccine and minimizing the risk of complications.

Lastly, as this is an institutional-based study, the findings may not reflect the true prevalence of BCG complications in the general population. A population-based study in countries with national BCG vaccination programs would be needed to better address this issue.

## Conclusion

In summary, severe local complications from the BCG vaccine or disseminated BCG infection should be regarded as primary indicators of an underlying IEI. In such cases, physicians should promptly refer patients for comprehensive immune system evaluations. A multi-faceted approach including neonatal IEI screening (TREC, KREC), delayed BCG vaccination, targeted immunological testing, and vaccination protocols tailored to country-specific conditions, is essential to optimize patient outcomes and minimize BCG-related complications in individuals with IEI.

## Data Availability

The raw data supporting the conclusions of this article will be made available by the authors, without undue reservation.
